# Crossover design in triage education: the effectiveness of simulated interactive vs. routine training on student nurses’ performance in a disaster situation

**DOI:** 10.1186/s13104-023-06596-5

**Published:** 2023-11-05

**Authors:** Mohsen Masoumian Hosseini, Seyedeh Toktam Masoumian Hosseini, Karim Qayumi, Shahriar Hosseinzadeh, Soleiman Ahmady

**Affiliations:** 1 Department of E-learning in Medical Sciences, Tehran University of Medical Sciences, Tehran, Iran; 2 CyberPatient Research Affiliate, Interactive Health International, Department of the surgery, University of British Columbia, Vancouver, Canada; 3 Department of Nursing, School of Nursing and Midwifery, Torbat Heydariyeh University of Medical Sciences, Torbat Heydariyeh, Iran; 4 Professor at Department of Surgery, University of British Columbia, Vancouver, Canada; 5 CyberPatient Research Coordinator, Interactive Health International, Department of Surgery, University of British Columbia, Vancouver, Canada; 6 Department of Medical Education, Virtual School of Medical Education & Management, Shahid Beheshti University of Medical Sciences, Tehran, Iran; 7 Department of LIME, Research Affiliated Faculty, Karolinska Institute, Solna, Sweden

**Keywords:** Gamification, Triage, Simulated Interactive Guideline, Training, Emergency medicine, Crossover study

## Abstract

**Introduction:**

This study investigates the effectiveness of incorporating simulated interactive guidelines in nursing students’ performance during disaster situations, compared to routine training.

**Method:**

This study was a crossover design with pre-and post-tests for two groups. Each group consisted of 60 students selected using the census method. SIG and routine (Face-to-Face) training sessions were conducted as a crossover design. Triage knowledge questionnaires were used in the pretest to assess triage knowledge. An OSCE test was administered in the posttest to assess student performance, followed by a triage skills questionnaire. Both questionnaires were highly reliable, as indicated by Cronbach’s alpha coefficients (0.9 and 0.95, respectively). Statistical analysis was performed using SPSS version 26 software at a significance level 0.05.

**Result:**

The chi-square test showed that the two groups were homogeneous regarding age. Regarding knowledge level, both groups were homogeneous before the intervention (P = 0.99). Nevertheless, the results of the OSCE test showed that the students in Group A had a higher level of skill than the students in Group B (93% versus 70%). Also, 18% of the students in group B had low skills.

**Discussion:**

The study found that student outcomes improved in both groups receiving SIG, suggesting that interaction and simulation improve learning. However, gamification is an ideal precursor to learning and not a substitute for education. Therefore, gamification should not be used as a stand-alone teaching method.

**Conclusions:**

The crossover study found that simulators and games should not be considered stand-alone teaching methods but can contribute to learning sustainability when used alongside instruction.

**Supplementary Information:**

The online version contains supplementary material available at 10.1186/s13104-023-06596-5.

## Introduction

Emergency Medicine students must understand disaster triage principles to effectively prioritize patients and optimize medical resources during increasing disasters, as incorrect practices can hinder disaster prevention efforts [1–3]. Triage systems are developed for Mass Casualty Incidents (MCI) to allocate limited medical resources appropriately, prioritizing individuals in need of emergency treatment to reduce mortality and morbidity rates [4, 5].

The US, the UK, Australia, and Iran have implemented various triage methodologies in disaster situations. However, there is no universally recognized and definitive gold standard for a triage system [6]. The Simple Triage and Rapid Treatment system (START) is the most widely used in the US, followed by Triage Sieve in the UK and CareFlight Triage in Australia [6]. A modified version called JumpSTART is used for pediatric patients [7]. In Iran, adults and children aged one year and above are assessed using the Triage START/JumpSTART system after accidents or high casualties [8]. The START process, a color-coding system for categorizing patients based on physiological factors, is used for disaster decision-making [9, 10]. Triage accuracy ranges from 64 to 90% in simple training but drops to 50–80% in realistic simulated environments [11, 12]. The time required to triage simulated patients varies between studies. In a simulated MCI with JumpSTART, triage took 23 s, while a multiple casualty exercise with START took over twice as long [13, 14]. The stress and rarity of real-life emergencies can make remembering proper steps difficult [15].

Studies reveal a significant knowledge gap in nursing students about triage, with insufficient education and training in countries like Sweden [16], Iran [17] and Australia [18]. In Australia, 42% of nurses lack formal triage training, and 14% report insufficient preparation [18]. This discrepancy is further compounded by the limited exposure emergency medicine students receive during a mere two-hour session on triage education within Iranian healthcare institutions [19]. The Iranian Ministry of Health has introduced guidelines making triage education compulsory for all nurses and emergency medicine technicians, aiming to improve nurses’ skills in assessing and prioritizing patients [20]. Healthcare professionals must develop effective educational approaches to enhance nursing students’ decision-making abilities in triage situations [21].

Gamification in medical education has become popular due to its ability to enhance cognitive abilities like analytical thinking, spatial reasoning, and memory retention. Studies show that gaming also improves motor dexterity and spatial awareness [22–24]. A study by Jalink et al. found a positive correlation between computer gaming proficiency and improved performance in endoscopic simulation tasks among medical students [25]. De Lisi and Wolford (2002) found that playing games like Tetris can improve spatial abilities [26]. Triage training uses both in-person and virtual simulations [27]. In-person simulations are most effective but require a large amount of resources, including supplies and personnel [27, 28]. Virtual simulations can be an effective tool for teaching disaster triage principles in resource-limited environments [29]. This study aims to evaluate the effectiveness of incorporating simulated interactive guidelines instead of routine training in optimizing nursing students’ performance in disaster situations. This approach could help reduce logistical challenges and improve the overall training experience.

## Materials and methods

**Fig. 1:**
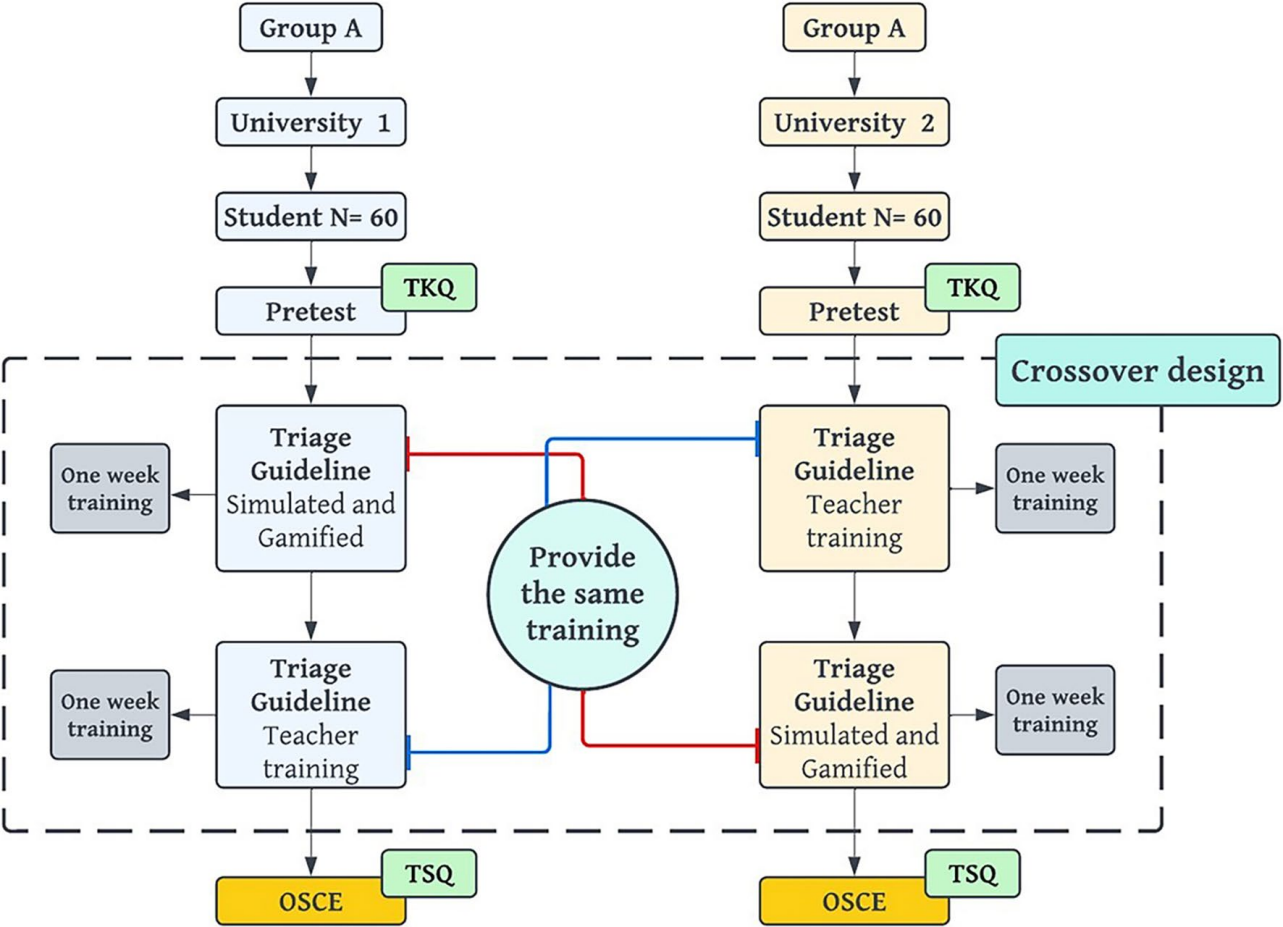
Crossover design process. Both groups received training that was coordinated in terms of timing and content

A quasi-experimental repeated measures 2 × 2 cross-over design was used to compare the effectiveness of Face-to-Face teacher training and Simulated Interactive Guideline (SIG) in two equally ranked universities. The groups were presented with different teaching methods, with group A (NUMS) receiving the SIG first and then Face-to-Face teacher training. In group B (THUMS), the process was reversed, resulting in the same training but on a different schedule. An overview of the design of the study can be found in Fig. 1. All learners voluntarily consented, and the Virtual University of Medical Sciences ethics committee approved the study.

### Design SIG

**Fig. 2:**
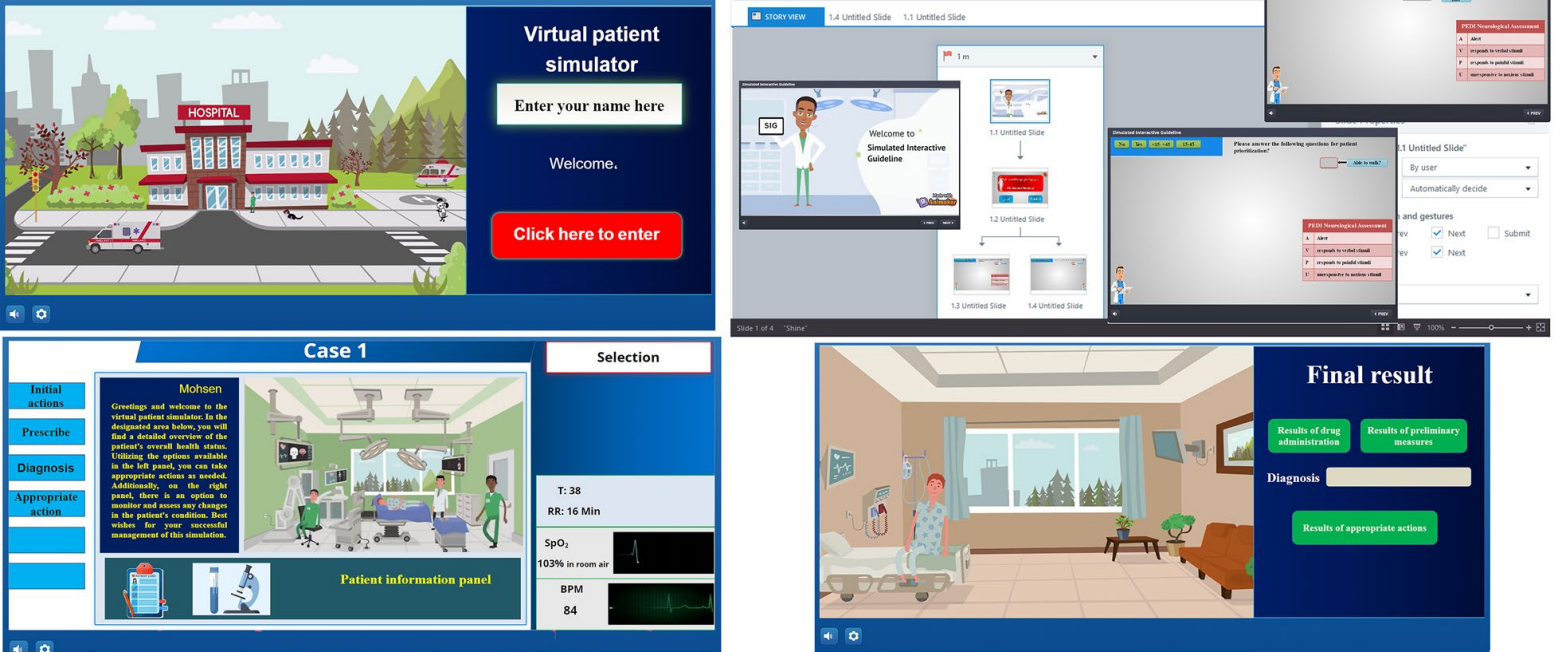
SIG environment screenshots

A team of experts from e-learning, medical education, and triage developed SIG, a non-linear simulation tool. The graphic interface was designed using Articulate Storyline 3 and a university’s learning management system. SIG was developed using START and JumpSTART guidelines, offering different scenarios based on a patient’s condition. There were no preset scenarios, allowing learners to create their own scenarios. The voice-activated user interface reviewed the scenarios and announced the results. The images of the virtual environment from SIG can be seen in Fig. 2, and the video presentation of the scenarios is at the link bit.ly/3Je6rcj. Readers can access the created scenarios via the link https://cloud.scorm.com/sc/guest/SignInForm and test their knowledge by entering the username “masoumian.m@vums.ac.ir” and the password “vums”. Enter the course “Simulated Interactive Guideline”, then click on " launch course” and use SIG.

### Sample and size

Study participants were selected from two universities using the census method (N = 120) and divided into groups A and B randomly. Each group belonged to one university (N = 60). Before starting the intervention, the researcher visited the two groups’ training sites and explained SIG and face-to-face teaching to the students. Eligibility for participation in the intervention was extended to emergency medicine students who were in their third semester and possessed reasonable computer skills. It is often assumed by instructors that this particular generation of students, having grown up surrounded by technology, would naturally be proficient with computers [30, 31]. A self-reported questionnaire was administered to evaluate their aptitude towards information and communication technologies. Participants who were willing to participate in the intervention, able to attend the face-to-face classes, and who did have adequate computer skills were included in the study.

### Pre-intervention

The Triage Knowledge Questionnaire (TKQ) was used to assess students’ knowledge of triage before an intervention, consisting of ten questions on triage and priority categories for injured patients in crisis situations [32]. The test had a correlation coefficient of 0.99.

### Intervention

Each intervention was assigned a teacher. Thus, both groups were taught by the same teachers.

#### Group A

**Fig. 3:**
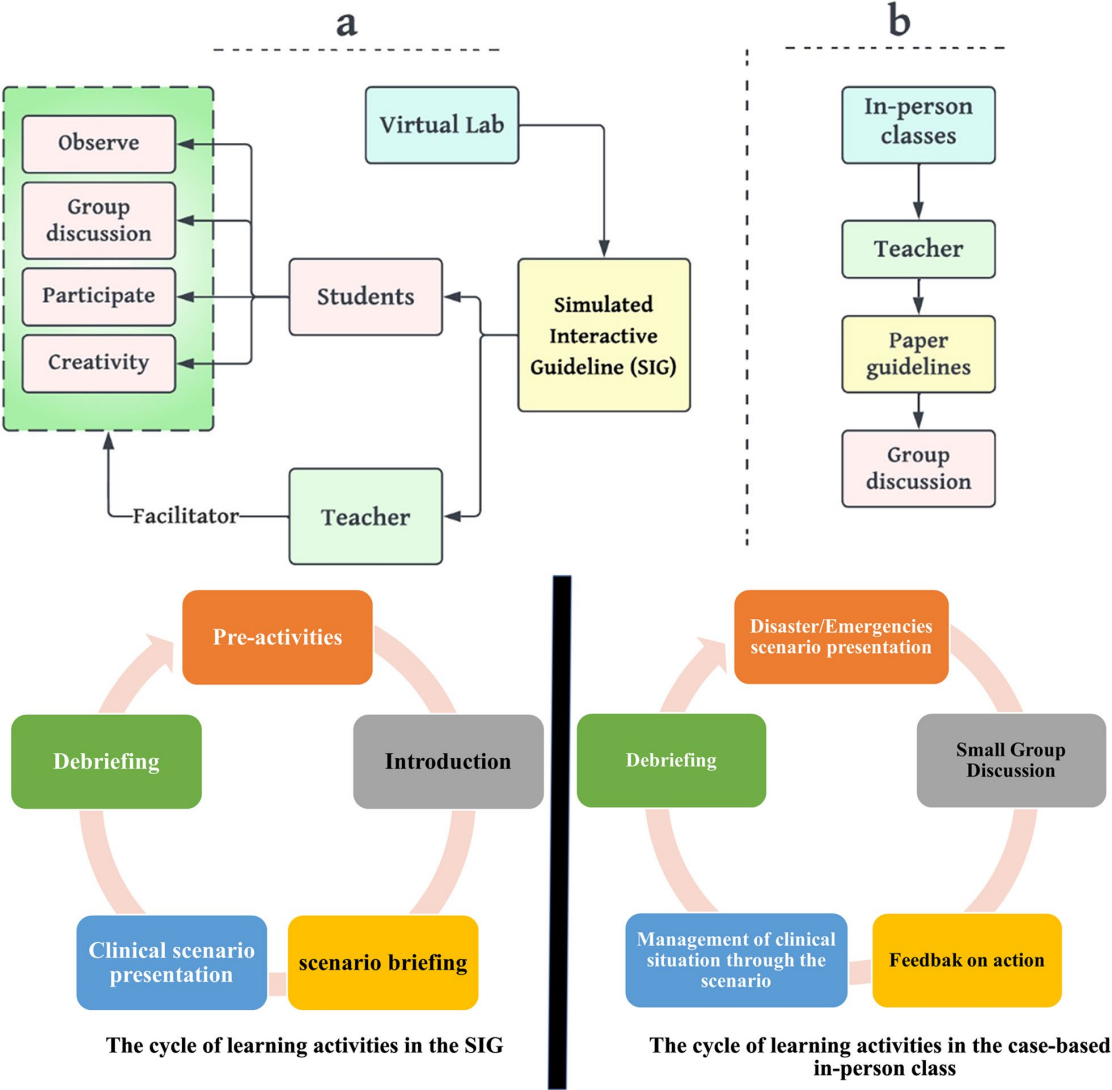
Crossover study activities. **a** Teacher training; The teacher discussed the paper’s guidelines in groups during each session. **b** SIG training; During scenario creation, the teacher facilitated learning and students learned by creating different scenarios

The school of nursing organized a comprehensive training program for students, covering nursing principles in emergencies and disasters. The course covered aspects such as providing effective solutions, implementing nursing interventions triage procedures, and understanding the role of nurses in patient triage. It also focused on providing high-quality pre-hospital care using a decision-making priority process. The course also covered didactic hazard vulnerability analysis techniques and incident command system protocols. Students were exposed to concepts like incident facilities management, disaster capacity framework examination, and efficient triaging methods [33, 34]. The SIG training was conducted for five days for group A students, with a teacher acting as a supervisor and facilitator. Students were introduced to the SIG and provided detailed information about its functions. They were also given 10 min to familiarize themselves with the game and completed a demographic information questionnaire. The educational strategy of case-based learning was used, involving pre-activities, introducing the subject matter, scenario briefing, engaging in game-based disaster/emergency scenarios, and culminating with presentation and debriefing sessions (see Fig. 3a).

##### Pre-activities

Students were introduced to 25 case studies through a virtual classroom dashboard in the university’s learning management system (Additional file 1: Appendix S1). A competitive element was introduced by displaying participants’ results as virtual avatars in the LMS while ensuring the confidentiality of individual information. The intervention took place over a period of five days spanning three weeks at the university clinical laboratory, with students engaging in practical exercises on triage, disaster response, and emergencies. Each exercise involved analyzing and addressing specific cases guided by a clinical professor.

##### Introduction

The clinical instructor discussed educational goals for working with the virtual patient Fig. 3b and inquired about the comprehensive prior knowledge relevant to this case during a discussion.

##### Scenario briefing

Following a concise introduction by the clinical professor, students promptly engaged with the virtual patient, immersing themselves in their hands-on learning experience.

##### Clinical scenarios presentation

A one-hour session was conducted with a virtual patient, starting with students logging in and receiving feedback from the instructor. Users were granted access through their usernames and passwords, and the interactive virtual patient experience included textual content, images, videos, and animations. The system was gamified and provided a comprehensive learning experience.

##### Debriefing

Students completed virtual patient’s work and had a 90-minute debriefing session at the hospital conference hall. A clinical professor conducted the sessions, using a 3D model for comprehensive assessment. Five days later, face-to-face training was conducted for this group, followed by a week for analysis and a posttest. The students followed INACSL standards for comprehensive assessment and analysis.

#### Group B

Group B students underwent five days of face-to-face training with the same instructor who taught group A. The pedagogical approach involved in-person instruction, presenting practical scenarios related to emergencies and disasters. These scenarios included simulated emergencies and strategic questions to meet learning objectives. To enhance problem-solving skills, students engaged in small-group discussions. The lecturer’s role shifted to facilitator, providing guidance and support. In subsequent stages, traditional classroom lectures were conducted by the same instructor, covering educational themes within SIG’s intervention framework. The lecture topics were closely related to the cases presented. The course focused on START and JumpSTART triage methodologies, focusing on emergency nursing in crises and unforeseen incidents. Students explored principles and core concepts for exceptional care during emergencies. They engaged in creative thinking, effective communication, and problem-solving skills in a simulated pre-hospital setting, gaining hands-on experience in navigating the triage process confidently and effectively.

The researcher conducted a formal debriefing session to understand participants’ triage levels and decision-making processes. They reviewed the START/JumpSTART algorithm and various case scenarios involving different age groups and acuity levels. The debriefing aimed to engage participants in thoughtful discussions, often addressing general pediatric and adult emergency care practices. After each session, the researcher read a standardized statement summarizing learning objectives, encouraging further questions. Participants were allowed to raise inquiries about simulated cases or information obtained through self-directed learning from external resources. Then, the training was conducted by SIG for five days with a trainer leading the learning, similar to the training for group A. Participants were then given one week to reflect on their learning, followed by a posttest. A comparison of the activities carried out by the two groups is shown in Fig. 3.

### Data collection tools

Triage skills were measured using the Objective Structured Clinical Examination (OSCE) with three stations (rapid assessment, categorization, and patient allocation) via the Triage Skills Questionnaire (TSQ) [32]. Experts from the field evaluated the content and validity of the instruments. During an orientation session one week before the OSCE, students received information about the OSCE process through written instructions explaining what would be expected of them. The experts recorded the students’ performance in the questionnaire during the test. This questionnaire includes 37 questions and tests three dimensions: rapid assessment, categorization, and patient allocation. In the study, experts were asked to rate on a rating scale of 1–5: 1 = needs improvement, 2 = poor, 3 = fair, 4 = good, and 5 = very good, with a total score ranging from 37 to 185; the final score was converted to a percentage ($\frac{Scores}{185} \times 100$). The final score was divided into low (60%), moderate (60–80%), and high (over 80%) triage skills. The triage skills questionnaire was highly reliable in the study, with Cronbach’s alpha coefficient of 0.95. To determine the validity of the native language version of the tests, the standard protocols of the International Quality of Life Assessment (IQOLA) were used [35]. This protocol consists of four steps: Translation, evaluation of translation quality, back translation, and comparison of the English version with the native language version. In addition to face validity, an impact score of 20 experts was analyzed. For each of the 37 items in this questionnaire, there were three parts in which difficulties in understanding concepts, ambiguity and misperceptions, and appropriateness and coherence were assessed separately. For simplicity and clarity of statements, an agreement rate of at least 80% was used for face validity (i.e., at least 16 people scored 4 and 5 out of 5 for the simplicity and clarity criteria). An agreement rate of at least 80% and a compelling value greater than 1.5 indicate that the item has good external validity and is perceived by participants as easy to understand simple to apply, meaningful, and fluid. The expert panel (researcher and four experts) revised and corrected the question if the effect value was less than 1.5.

Due to the importance of the topic, the students were divided into five groups and had thirty minutes to present their work. In total, the test took about seven hours. Similarly, the next day, the students in group B were taken to the location designated for their test, where the experts’ assessments were recorded. Participants in each group believed they were the only ones participating in the study.

### Analyzes

The study used SPSS version 26 software to analyze data with a significance level of 0.05. The Shapiro-Wilk test confirmed normal distribution, and independent t-tests were performed for between-group comparisons. The relationship between dependent and independent variables was assessed using percentage and frequency calculations, and adjusted odds ratios and chi-square tests were calculated. The data was presented in numbers, frequencies, tables, and graphs.

## Result

The purpose of the present study was to examine the effects of SIG on students’ ability to recognize and cope with disaster scenarios in ambulatory critical care at the two schools of nursing. The chi-square test revealed age homogeneity between groups A and B. In groups A and B, the mean age was 18.35 (0.8127) and 18.30 (0.9787), respectively, which was not a significant difference (P = 0.99). The TKQ scores of students in groups A (3.13 ± 1.54) and B (2.9 ± 1.55) were not significantly different (P = 0.412). Both groups were, therefore, homogeneous in their knowledge of triage.

### OSCE test

**Fig. 4:**
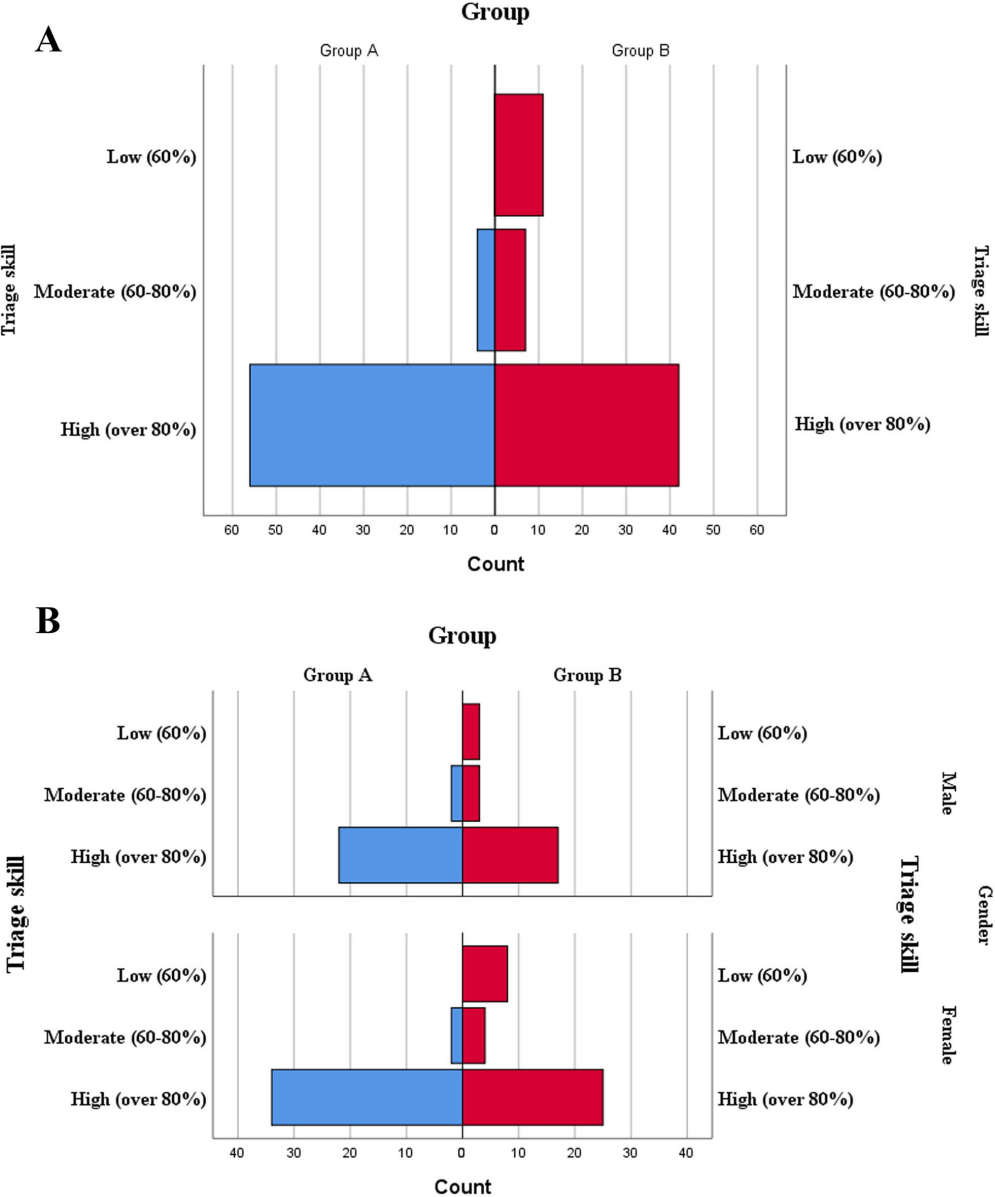
Comparison of OSCE results in two groups. Group **A** assesses the overall performance level, highlighting proficiency in three tiers: Low, Moderate, and high. Meanwhile, Group **B** examines gender-based disparities across these two groups

In reviewing the results of the OSCE test, it was found that 93% (56) of the students in group A had a high level of skill, of which 61% (34) were female and 39% (22) were male. In group B, 70% (42) of the students had a high level of triage skills, of which 59% (25) were female and 41% (17) were male. A moderate level was achieved by 7% of the students in group A, of whom 50% were female and 50% male. In group B, 12% of the students had moderate skills; 57% were female, while 43% were male. Of the students in group B, 18% had low skills, of which 73% were female and 27% were male (Fig. 4).

### OSCE test subscales

According to the results of the OSCE subscales for group A, 85.5% of the students performed very well on the Rapid Patient Assessment, while only 2% performed poorly, and 1.4% had to relearn. Likewise, the results of this subscale in group B showed that 68.4% of the students did very well, 7.8% did poorly, and 8.2% needed additional training. In group A, 95.4% of the students scored very well in the patient categorization subscale, and none needed retraining. Also, in this subscale, 81% of the students in group B scored very well, 5.7% scored poorly, and 1.2% needed retraining. According to group A’s patient assignment subscale results, 93.3% of the students performed very well, and 0.3% needed retraining. In group B, the results of this subscale showed that 76.4% of the students did well, 6.2% did poorly, and 4.7% needed retraining. Table 1 presents a more detailed description of each subscale’s results.

**Table 1:** The results of the OSCE test in both groups for each item and subscale

Triage skills		Perceived Triage Skill									
		Group A					Group B				
		V	G	F	P	N	V	G	F	P	N
No	**Rapid patient assessment**	85.5%	7%	4.1%	2%	1.4%	68.4%	7.8%	7.8%	7.8%	8.2%
1	A assess patient include vital signs with rapid assessment in 2–5 min	53 (88.3%)	4 (6.7%)	2 (3.3%)		1 (1.7%)	38 (63.3%)	9 (15%)	4 (6.7%)	2 (3.3%)	7 (11.7%)
2	A assess or ask chief complaint of the patient rapidly	51 (85%)	7 (11.7%)	1 (1.7%)	1 (1.7%)		34 (56.7%)	7 (11.7%)	6 (10%)	8 (13.3%)	5 (8.3%)
3	In unconscious patients, look in the upper airway such as blood, vomit, foreign bodies, oedema, and tongue obstruction as assess airway patency	49 (81%)	8 (13.3%)	3 (5%)			36 (60%)	5 (8.3%)	9 (15%)	7 (11.7%)	3 (5%)
4	Decide to open airway and remove foreign body when airway is obstructed according to airway management (A)	48 (80%)	3 (5%)	5 (8.3%)	3 (5%)	1 (1.7%)	41 (68.3%)	5 (8.3%)	3 (5%)	5 (8.3%)	6 (10%)
5	Give positioning airway to maintain patency by chin lift	57 (95%)	1 (1.7%)	2 (3.3%)			44 (73.3%)	2 (3.3%)	6 (10%)	7 (11.7%)	1 (1.7%)
6	Perform clear airway by correct position with jaw trust and head tilt	48 (80%)	2 (3.3%)	5 (8.3%)	3 (5%)	2 (3.3%)	39 (65%)	4 (6.7%)	3 (5%)	5 (8.3%)	9 (15%)
7	Perform clear airway by correct position by jaw trust without head tilt if the patient suspect cervical spinal	52 (86.7%)	4 (6.7%)	3 (5%)	1 (1.7%)		40 (66.7%)	3 (5%)	8 (13.3%)	3 (5%)	6 (10%)
8	Perform to insert oropharyngeal or nasopharyngeal airway	50 (83.3%)	9 (15%)		1 (1.7%)		37 (61.7%)	6 (10%)	7 (11.7%)	1 (1.7%)	9 (15%)
9	Look at the chest about patient chest abnormal movement	57 (95%)	1 (1.7%)	2 (3.3%)			50 (83.3%)	2 (3.3%)	5 (8.3%)	3 (5%)	
10	Assess rate and depth of respirations to observe (B) breathing rate and pattern rhythm with look and listen	47 (78.3%)	7 (11.7%)	2 (3.3%)	1 (1.7%)	3 (5%)	35 (58.3%)	3 (5%)	4 (6.7%)	6 (10%)	12 (20%)
11	Look at the patient skin to investigate for integrity, wounds, bruising, texture, and color	51 (85%)	4 (6.7%)	1 (1.7%)	3 (5%)	1 (1.7%)	43 (71.7%)	3 (5%)	6 (10%)	8 (13.3%)	
12	Listen the noise in the airway such as gurgling, snoring, and wheezing	58 (96.7%)	1 (1.7%)	1 (1.7%)			46 (76.7%)	2 (3.3%)	5 (8.3%)	5 (8.3%)	2 (3.3%)
13	Listen the silent or noisy breathing	56 (93.3%)	2 (3.3%)	2 (3.3%)			49 (81.7%)	1 (1.7 5)		4 (6.7%)	6 (10%)
14	Feel air blow from the patient with my cheek	53 (88.3%)	4 (6.7%)	2 (3.3%)		1 (1.7%)	46 (76.7%)	4 (6.7%)	5 (8.3%)	3 (5%)	2 (3.3%)
15	Administer oxygen therapy	55 (91.7%)	3 (5%)	2 (3.3%)			39 (65%)	5 (8.3%)	7 (11.7%)	1 (1.7%)	8 (13.3%)
16	Perform manual ventilation	53 (88.3%)	7 (11.7%)				45 (75%)	2 (3.3 5)	4 (6.7%)	6 (10%)	3 (5%)
17	Perform bag-valve-mask ventilations	47 (78.3%)	4 (6.7%)	5 (8.3%)	2 (3.3%)	2 (3.3%)	33 (55%)	8 (13.3%)	5 (8.3%)	7 (11.7%)	7 (11.7%)
18	Protect cervical spine when patient suspect fracture cervical with cervical collar	42 (70%)	8 (13.3%)	4 (6.7%)	2 (3.3%)	4 (6.7%)	35 (58.3%)	6 (10%)	4 (6.7%)	9 (15%)	6 (10%)
19	Check pulse rate and rhythm according circulation assessment (C)	55 (91.7%)	3 (5%)	1 (1.7%)	1 (1.7%)		46 (76.7%)	5 (8.3%)	8 (13.3%)	1 (1.7%)	
20	Assess of the capillary refill	57 (95%)	2 (3.3%)		1 (1.7%)		48 (80%)	3 (5%)	4 (6.7%)	2 (3.3%)	3 (5%)
21	Assess the temperature the patients	52 (86.7%)	4 (6.7%)	2 (3.3%)	1 (1.7%)	1 (1.7%)	49 (81.7%)	4 (6.7%)	3 (5%)		4 (6.7%)
22	Assess the patient with diaphoresis	56 (93.3%)	4 (6.7%)				40 (66.7%)	6 (10%)	4 (6.7%)	4 (6.7%)	6 (6.7%)
23	Perform chest compressions in critical condition of the patient	52 (86.7%)	3 (5%)	4 (6.7%)	1 (1.7%)		41 (68.3%)	5 (8.3%)	6 (6.7%)	3 (5%)	5 (8.3%)
24	Collaborative with physician to administer emergency drugs	43 (71.7%)	5 (8.3%)	5 (8.3%)	4 (6.7%)	3 (5%)	34 (56.7%)	6 (6.7%)	2 (3.3%)	10 (16.7%)	8 (13.3%)
25	Assess internal and external bleeding	54 (90%)	3 (5%)	3 (5%)			38 (63.3%)	8 (13.3%)	4 (6.7%)	7 (11.7%)	3 (5%)
26	Perform control blood loss appropriately to stop bleeding the patient	40 (66.7%)	6 (6.7%)	8 (13.3%)	4 (6.7%)	2 (3.3%)	40 (66.7%)	2 (3.3%)	5 (8.3%)	6 (6.7%)	7 (11.7%)
27	Collaborate resuscitation to provide appropriate intravenous fluid	48 (80%)	4 (6.7%)	2 (3.3%)	4 (6.7%)	2 (3.3%)	42 (70%)	5 (8.3%)	4 (6.7%)	4 (6.7%)	5 (8.3%)
**Patient categorization**		95.4%	3.8%	0.8%			81%	7.1%	5%	5.7%	1.2%
28	Catagorization the patient according to triage priority	59 (98.3%)	1 (1.7%)				51 (85%)	3 (5%)	4 (6.7%)	2 (3.3%)	
29	Identify the patient who require immediate care, urgent, and non urgent according to triage categorization	58 (96.7%)	2 (3.3%)				49 (81.7%)	5 (8.3%)	3 (5%)		3 (5%)
30	Avoid the condition of the patient with over-triage and under-triage	55 (91.7%)	3 (5%)	2 (3.3%)			46 (76.7%)	2 (3.3%)	5 (8.3%)	7 (11.7%)	
31	Initiation nursing intervention during triage categorization	57 (95%)	3 (5%)				48 (80%)	7 (11.7%)		5 (8.3%)	
**Patient allocation**		93.3%	3.3%	3.1%		0.3%	76.4%	7.2%	5.5%	6.2%	4.7%
32	Make a decision to allocate the patient with priority 1 (Resuscitation in ED) in the right place	58 (96.7%)	2 (3.3%)				49 (81.7%)	6 (10%)	3 (5%)	2 (3.3%)	
33	Make a decision to allocate the patient with priority 2 (Critical care in ED) in the right place	56 (93.3%)	3 (5%)	1 (1.7%)			47 (78.3%)	4 (6.7%)	7 (11.7%)		2 (3.3%)
34	Allocate make a decision to allocate to the patient with priority 3 in the right place (Ambulatory in ED) correctly	57 (95%)		2 (3.3%)		1 (1.7%)	48 (80%)	3 (5%)	5 (8.3%)	2 (3.3%)	2 (3.3%)
35	Allocate the patient with nursing intervention safety in ED	54 (90%)	3 (5%)	3 (5%)			39 (65%)	7 (11.7%)	3 (5%)	8 (13.3%)	3 (5%)
36	Allocate the patient by collaboration with other emergency nurse & medical doctor with hand over effectively	58 (96.7%)	1 (1.7%)	1 (1.7%)			45 (75%)	2 (3.3%)		5 (8.3%)	8 (13.3%)
37	Allocate the patient to get advance treatment in ED in accurately and timely	53 (88.3%)	3 (5%)	4 (6.7%)			47 (78.3%)	4 (6.7%)	2 (3.3%)	5 (8.3%)	2 (3.3%)

V = Very good (5), G = Good (4), F = Fair (3), P = Poor (2), N = Need improvement (1)

## Discussions

This study has two main findings. First, the study showed that SIG based on gamification is anArticle structure: Kindly check whether the section headings have been identified correctly and amend if any. ideal precursor to learning, not a substitute for education. This result is confirmed by comparing the student’s scores on the OSCE test in the two groups. The test results revealed that group A’s interventions had a more significant educational impact than group B’s. In group A, SIG (learning content) was used as a precursor, and in group B, as a learning supplement. Numerous academic studies have been conducted to explore optimal methods for presenting learning content, particularly in the context of videos. Remarkably, these empirical investigations consistently indicate that incorporating video material prior to the primary instructional session yields significantly superior educational outcomes [36–38]. An earlier study by the authors examined the effects of teaching knowledge through gamification to nursing students using a standardized patient. It showed that gamification could improve students’ behavioral skills in disaster management [39].

Gamification can improve cognitive load and student performance, but it may increase extraneous cognitive load [40]. Research shows that gamification, when developed based on multimedia principles, can reduce cognitive load associated with subject matter and be used in conjunction with other teaching methods [41]. Incorporating game elements into multimedia learning helps learners maintain attention and concentration for extended periods [42]. Gamification can create lively interactions and motivate students to learn, providing a non-threatening environment for clinical courses [43, 44]. It promotes self-efficacy and allows students to learn and master problem-solving skills at a time, place, and pace [45, 46]. However, non-game-based learning may lead to boredom and lack of interest among learners [47, 48].

The study also found that student outcomes improved in both groups that received SIG, suggesting that interaction and simulation enhance learning. However, the temporal effects of this learning are unclear. This study has shown that learning presented before face-to-face instruction and as a precursor may prepare learners for the actual training and improve their understanding. There is growing evidence that face-to-face teaching is beneficial because of the interaction between learners and teachers and can fill many knowledge gaps [49]. The integration of Simulation and Imagination Games in educational settings offers students a unique opportunity to deeply immerse themselves in the subject matter [50]. By using SIG, educators are able to create interactive scenarios that encourage active student participation and facilitate a more comprehensive understanding of the content. The concept of gamification further enhances this learning experience by providing learners with an engaging environment where they can fully engage with the material at hand [44]. The study revealed significant levels of engagement in all groups, albeit with distinct variations observed between the different groups. Notably, Group A demonstrated heightened engagement right from the commencement of the lesson, whereas Group B experienced a surge in engagement subsequent to receiving instructions from the teacher. This interaction pattern began early on within Group A and persisted throughout their training under the instructor’s guidance. It is plausible that these divergent patterns account for group B’s comparatively lower scores when assessed via OSCE examination. Moreover, prior research has highlighted that deficient interactions can potentially impede individual content retention [51].

## Limitations

Despite efforts, the internet bandwidth between two universities was incompatible, causing the instructor to provide offline SIG support. Despite similar rankings, group B provided better academic support, adjusted by pretest. The same instructor taught at different distances, resulting in slightly longer lecture gaps, potentially affecting student education.

### Ethical issues

The study discusses students’ concerns about using a simulator, primarily due to limitations on smartphone access. Students expressed a desire for more time and a sense of confidence, arguing that the simulator could instill a false sense of safety. The researchers addressed this issue by using cross-over designs and teacher training.

## Conclusions

Both interventions increased student learning, but using the game at the beginning and subsequent teacher training was more effective, suggesting that games are better as antecedents. In group B, the game was used as a follow-up for teaching. This crossover study found that simulators and games should not be considered stand-alone teaching methods and that games contribute to learning when used in conjunction with instruction.

### Supplementary Information

**Additional file 1.** **Appendix 1.** Twenty-five meticulously crafted scenarios that served as the foundation for conceptualizing and bringing to life the immersive gaming experience.

## Data Availability

Upon reasonable request, the corresponding author can provide the data set that was analyzed during this study.

## Abbreviations

MCI: Mass Casualty Incident
EM: Emergency medicine
SIG: Simulated Interactive Guideline
IQOLA: International Quality of Life Assessment
OSCE: Objective Structured Clinical Examination
NUMS: Neyshabur University of Medical Sciences
THUMS: Torbat Heydarieh University of Medical Sciences
TSQ: Triage Skills Questionnaire
TKQ: Triage Knowledge Questionnaire
START: Simple Triage and Rapid Treatment System
EMS: Emergency Medicine Services
