# Supplementary material for: Crossover design in triage education: the effectiveness of simulated interactive vs. routine training on student nurses’ performance in a disaster situation
Source: BMC Res Notes. 2023 Nov 5;16:313. doi: 10.1186/s13104-023-06596-5 (PMC10626668; doi:10.1186/s13104-023-06596-5)
Supplement: Supplementary file 1 — Additional file 1. Appendix 1. Twenty-five meticulously crafted scenarios that served as the foundation for conceptualizing and bringing to life the immersive gaming experience. [file 13104_2023_6596_MOESM1_ESM.docx]

**Appendix 1.** Twenty-five meticulously crafted scenarios that served as the foundation for conceptualizing and bringing to life the immersive gaming experience

| 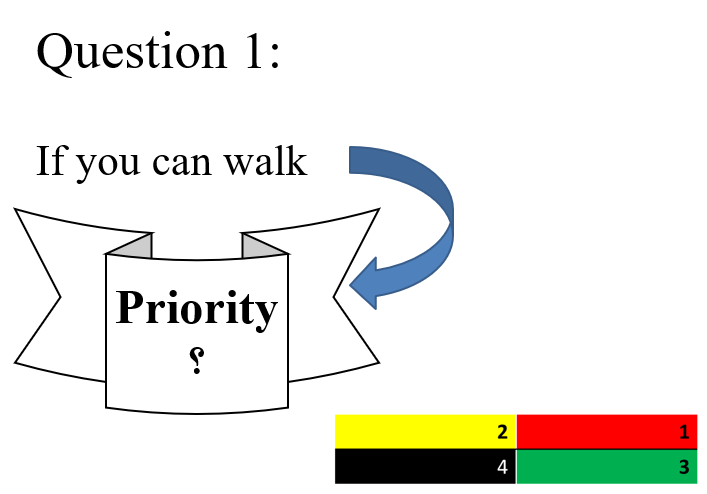 | 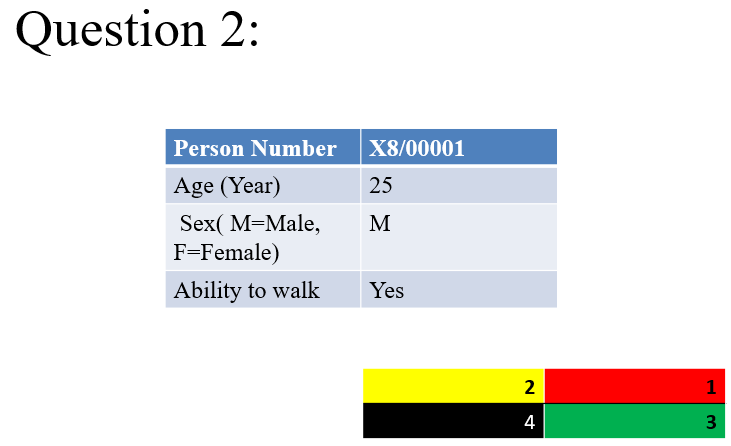 |
| --- | --- |
| 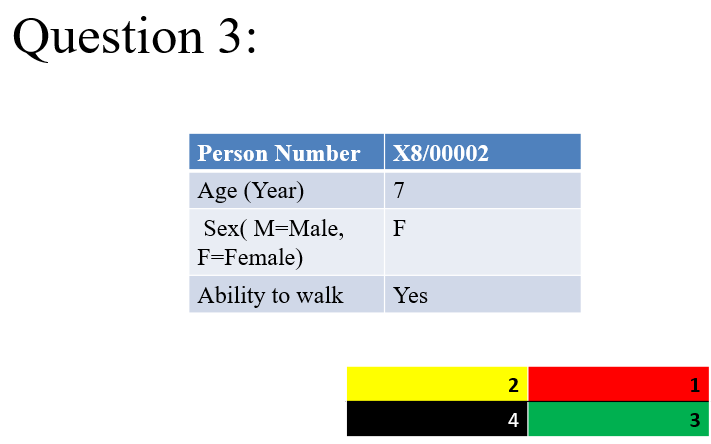 | 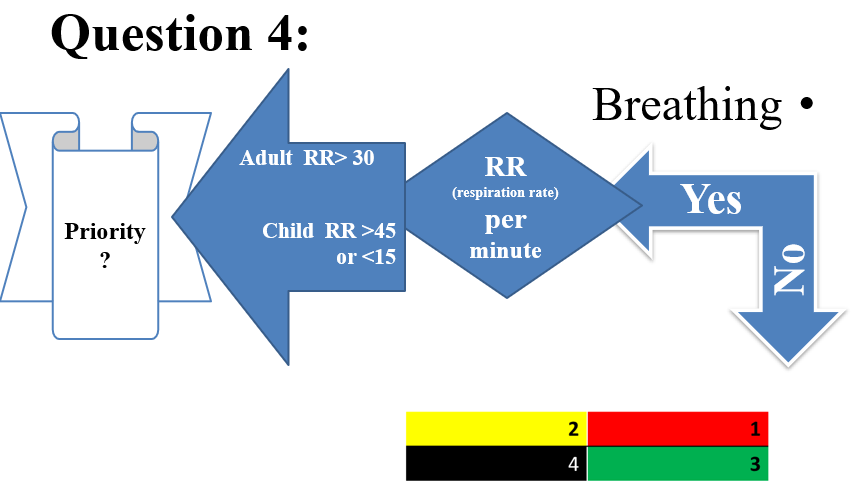 |
| 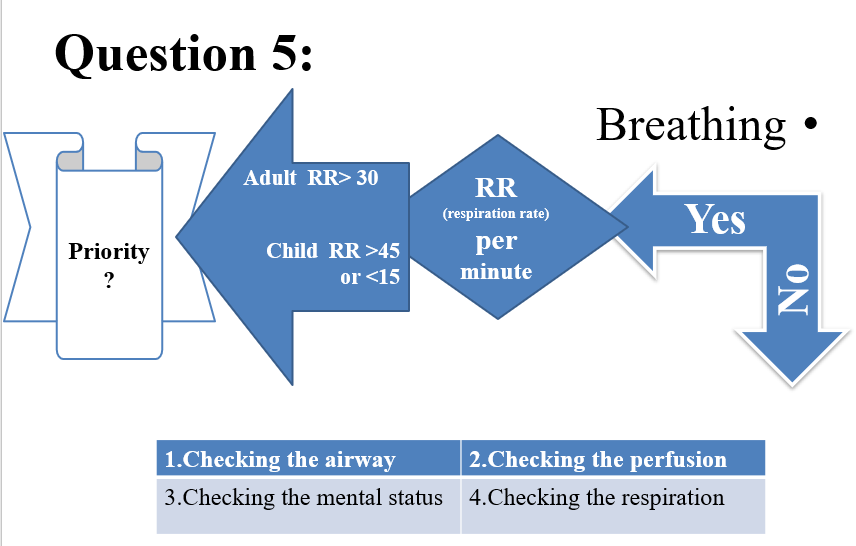 | 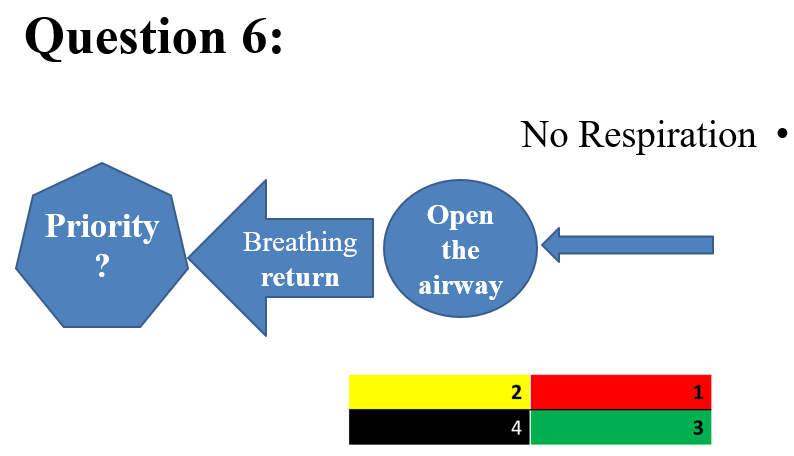 |
| 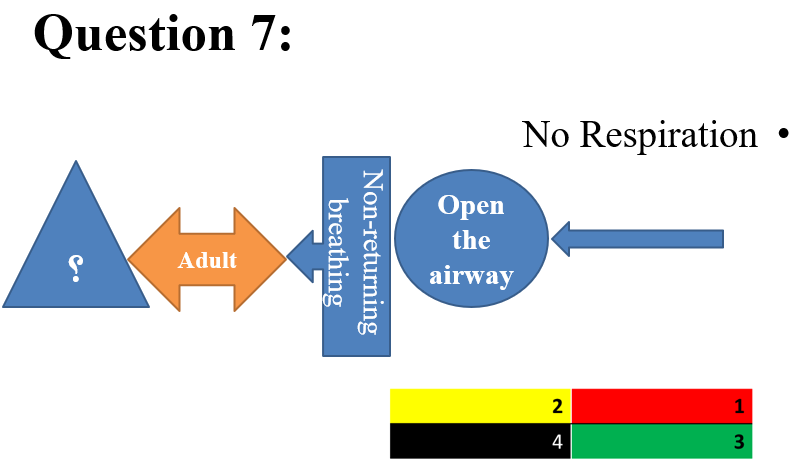 | 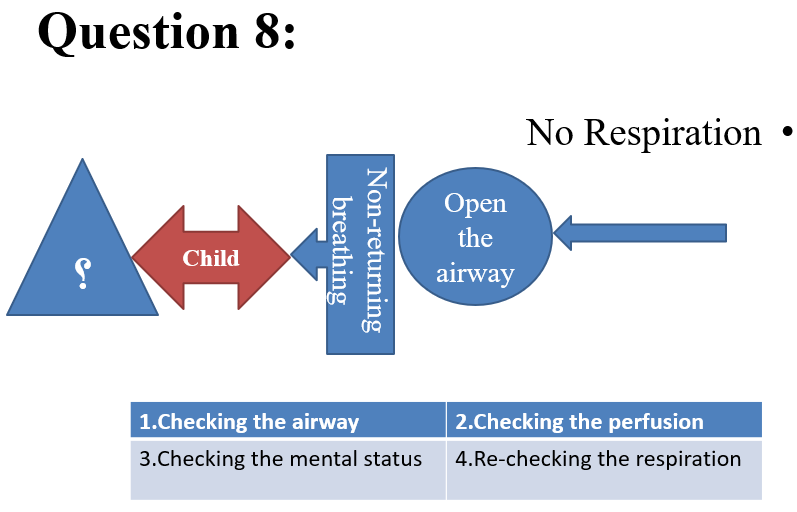 |
| 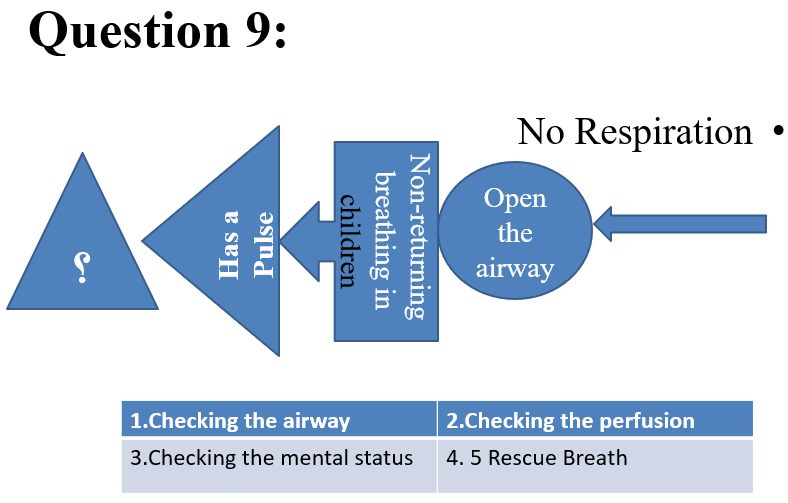 | 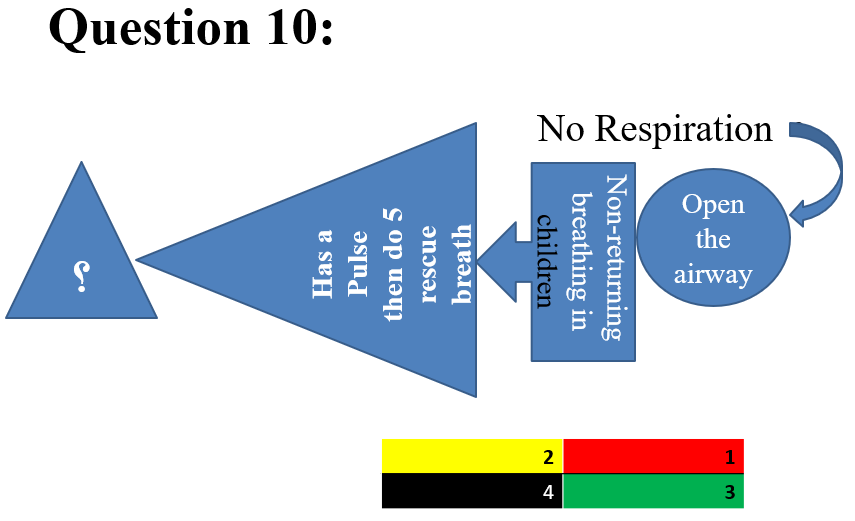 |
| 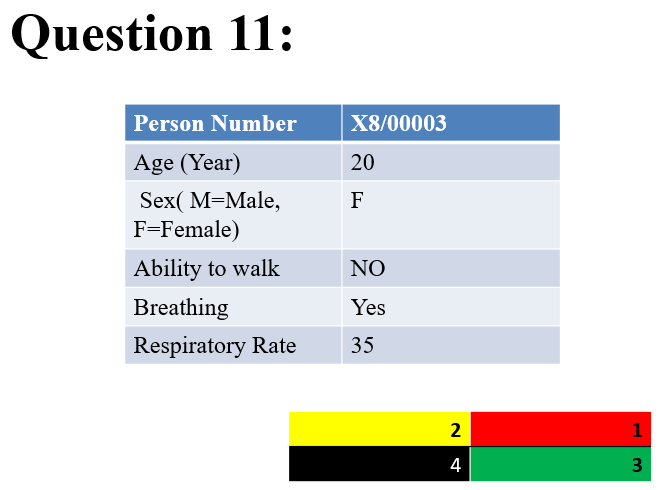 | 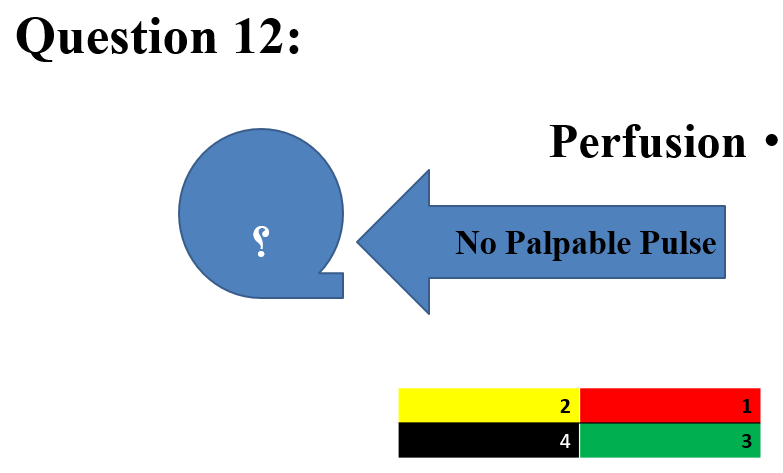 |
| 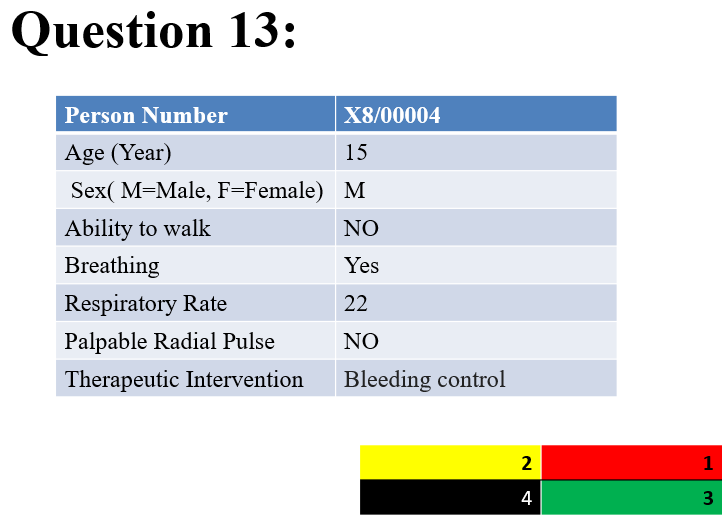 | 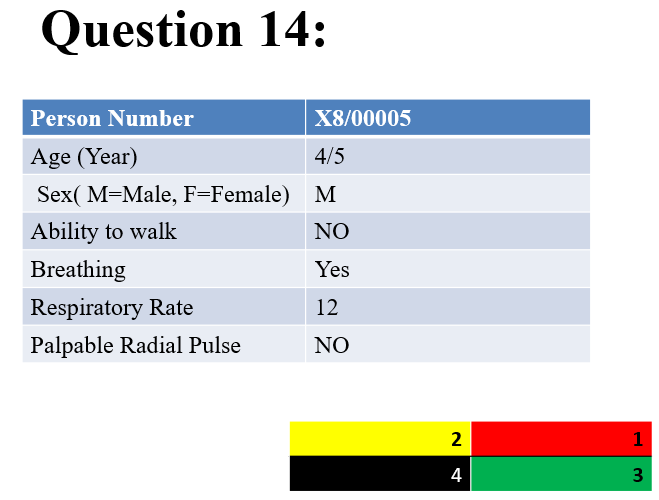 |
| 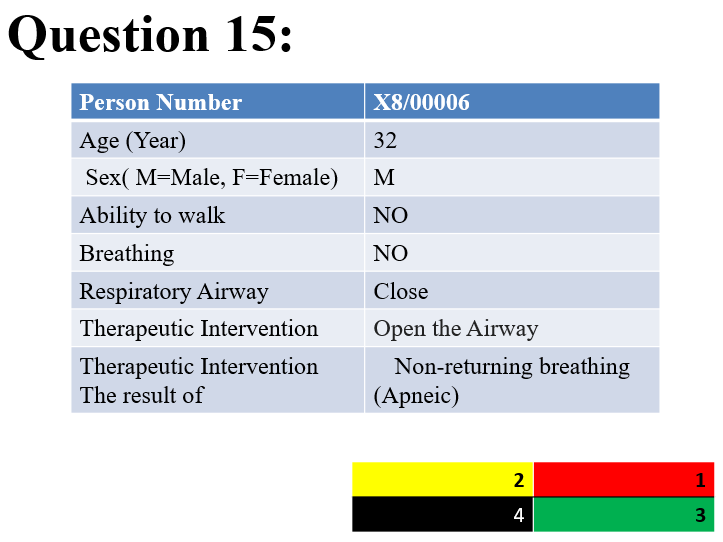 | 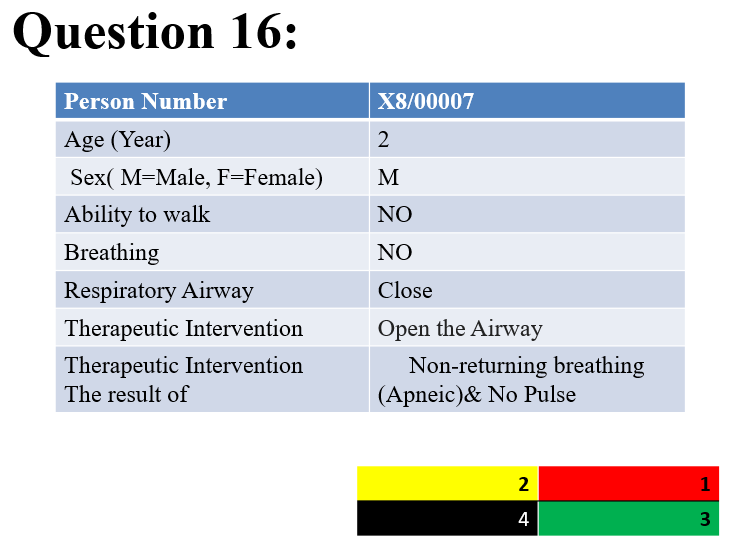 |
| 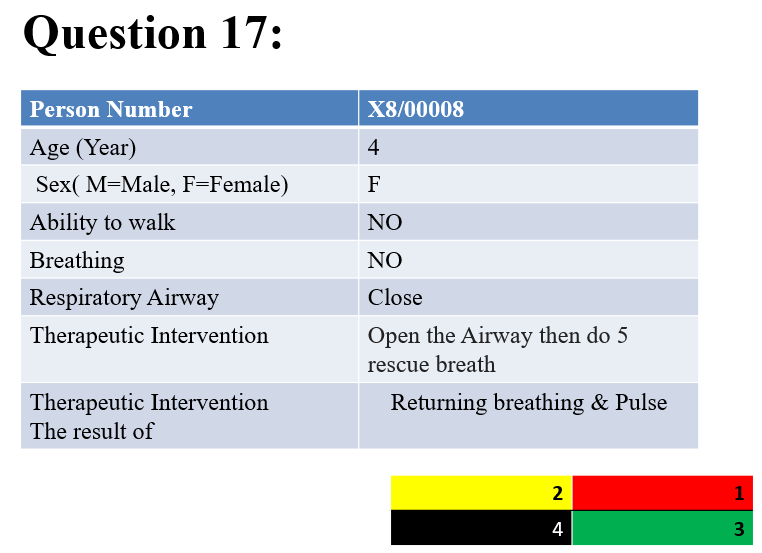 | 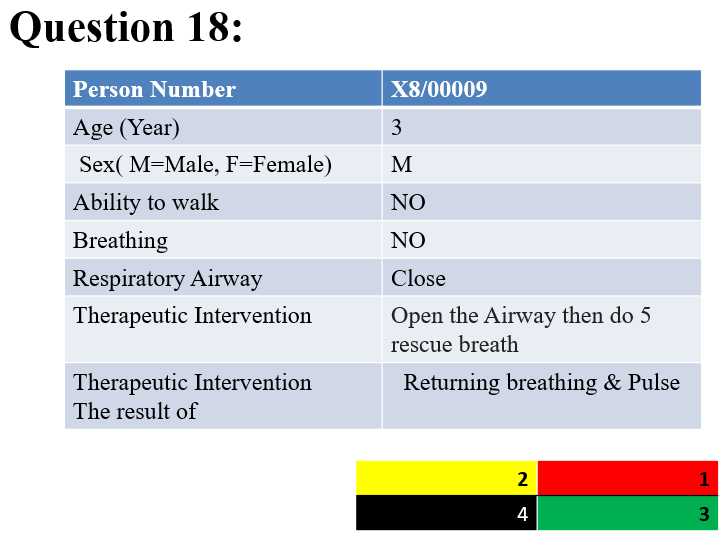 |
| 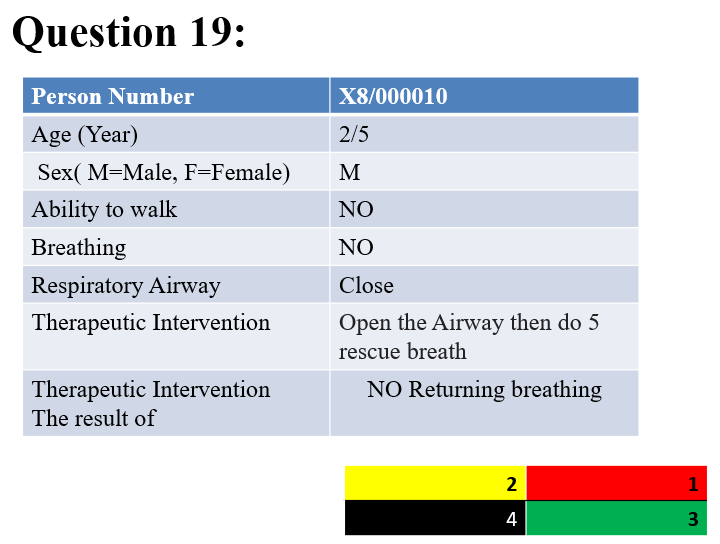 | 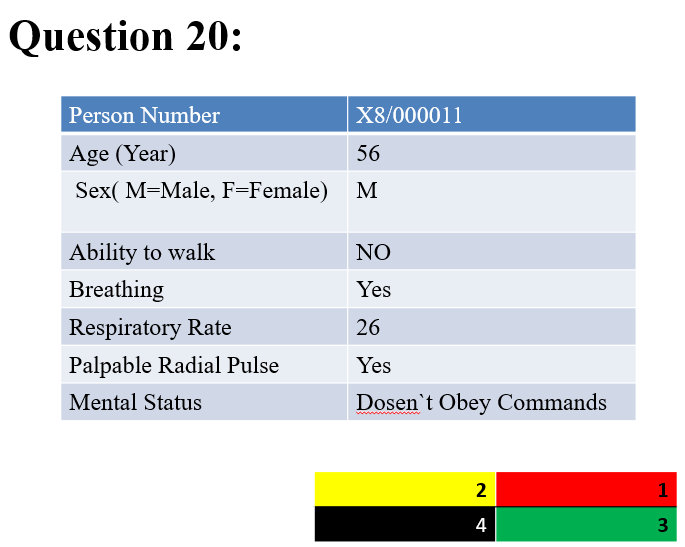 |
| 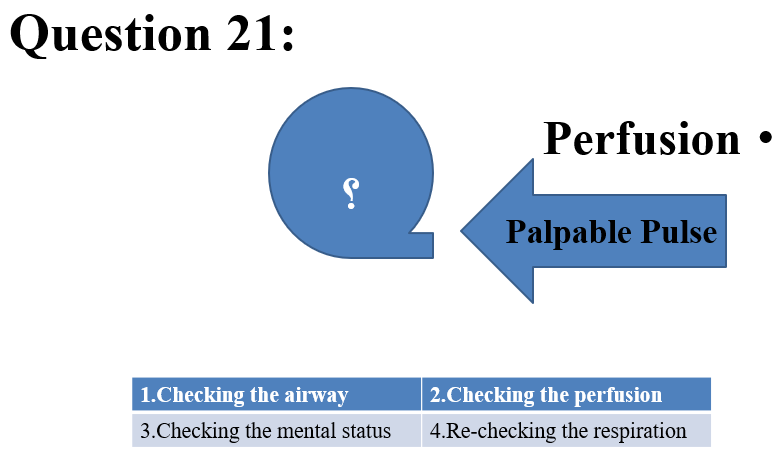 | 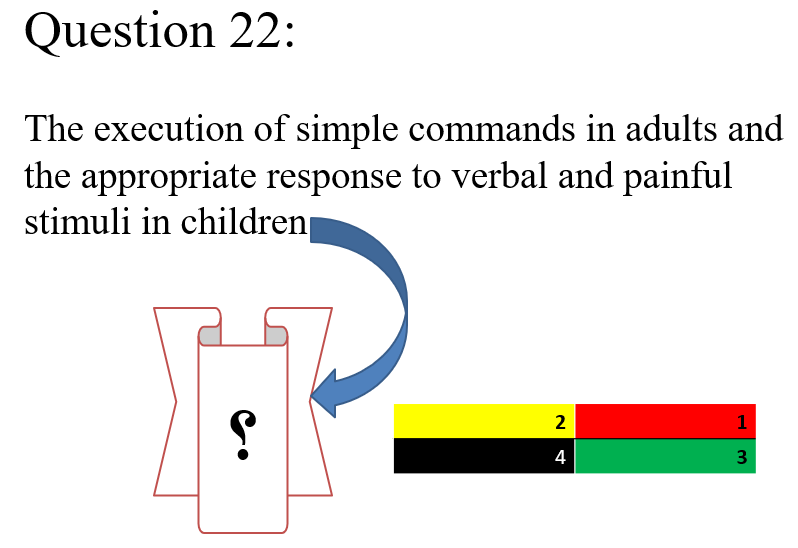 |
| 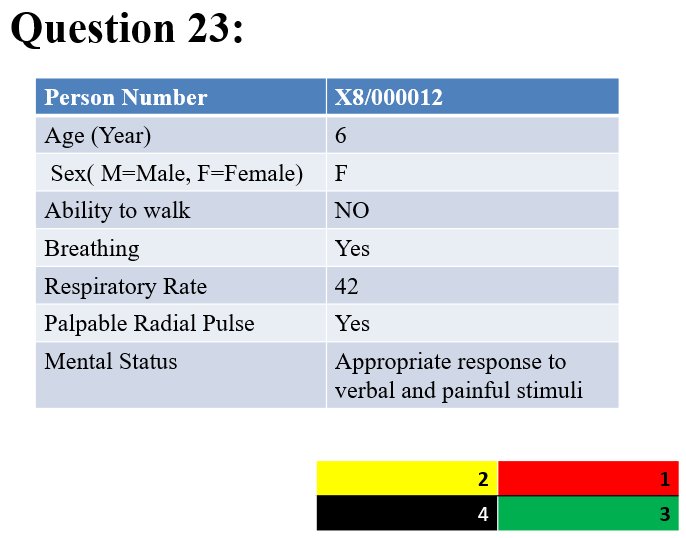 | 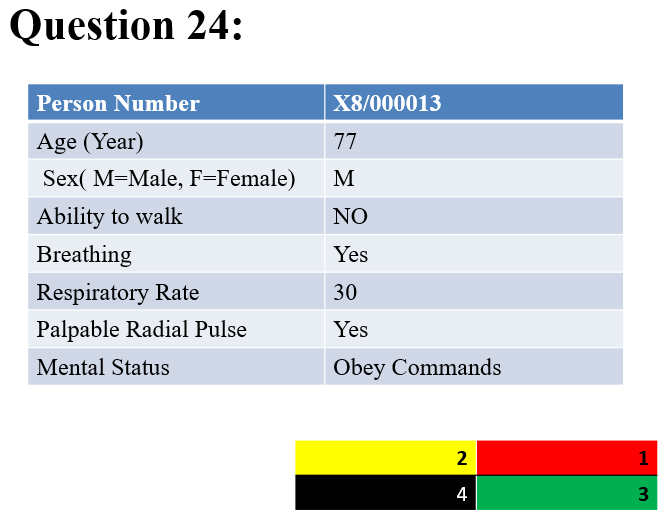 |
| 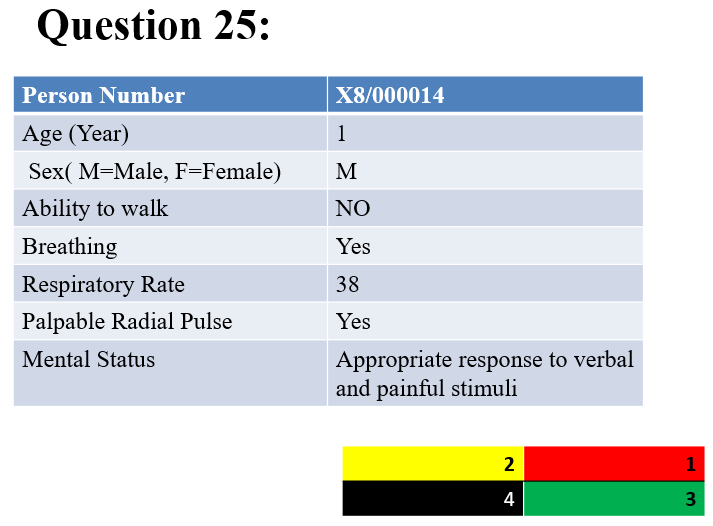 | 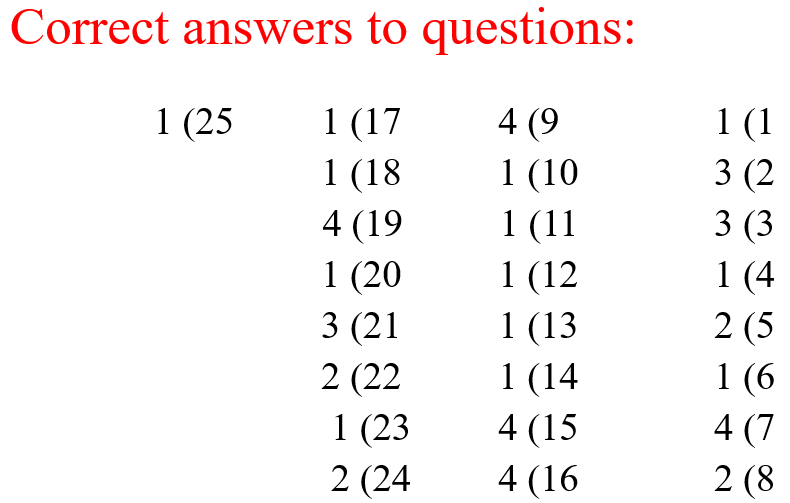 |
